# Active Vision Therapy for Anisometropic Amblyopia in Children: A Systematic Review

**DOI:** 10.1155/2020/4282316

**Published:** 2020-03-23

**Authors:** Carlos J. Hernández-Rodríguez, David P. Piñero

**Affiliations:** ^1^Group of Optics and Visual Perception, Department of Optics, Pharmacology and Anatomy, University of Alicante, Alicante, Spain; ^2^Clinical Optometry Unit, Department of Ophthalmology, Vithas Medimar International Hospital, Alicante, Spain

## Abstract

**Purpose:**

The aim of the study was evaluation of the scientific evidence about the efficacy of vision therapy in children and teenagers with anisometropic amblyopia by performing a systematic literature review.

**Methods:**

A search was performed using 3 searching strategies in 4 different databases (PubMed, Web of Science, Scopus, and PruQuest). The quality of the included articles was evaluated using two tools for the risk of bias assessment, ROBINS-I for nonrandomized studies of intervention (NRSI), and ROB 2.0 for randomized clinical trials.

**Results:**

The search showed 1274 references, but only 8 of them passed the inclusion criteria after the complete text review. The articles that were finally included comprised 2 randomized control trials and 6 nonrandomized studies of intervention. These articles provided evidence supporting the efficacy of vision therapy for the treatment of anisometropic amblyopia in children and teenagers. Assessment of the risk of bias showed an appropriate risk of bias for the randomized control trials, but a high risk of bias for nonrandomized studies of intervention (NRSI). A main source of risk of bias for NRSI was the domain related to the measurements of the outcomes, due to a lack of double-blind studies.

**Conclusion:**

Vision therapy is a promising option for the treatment of anisometropic amblyopia in children and teenagers. However, the level of scientific evidence provided by the studies revised is still limited, and further randomized clinical trials are necessary to confirm the results provided to date and to optimize the vision therapy techniques by knowing the specific neural mechanisms involved.

## 1. Introduction

Functional amblyopia is a visual developmental disorder consisting of reduced best-corrected visual acuity in one or rarely in both eyes without the presence of any ocular pathology [1]. Due to the abnormalities in visual processing occurring in amblyopia, there are also deficits in contrast sensitivity, accommodation, binocular vision, fixation, saccades, color, and form and motion perception, among others [2–9]. There are four types of amblyopia depending on its etiology: anisometropic amblyopia, strabismic amblyopia, mixed amblyopia (if anisometropia and strabism coexist), and deprivation amblyopia (if there was pathology during visual development which struggled the eye stimulation) [7]. The prevalence of amblyopia in childhood is approximately between 1 and 3%, although these values differ among authors [10, 11].

All types of amblyopia affect the primary visual cortex and extra-striate visual cortex (V1 and V2 areas, respectively), but magnetic resonance studies have shown that anisometropic amblyopia is also associated with decreased values in fractional anisotropy in the optic radiation, right superior longitudinal fasciculus, and inferior longitudinal fasciculus/inferior fronto-occipital fasciculus. In addition, increased fractional anisotropy values have been detected in the right posterior part of the corpus callosum [12]. On the other hand, strabismic amblyopia has been found to be associated with poorer functional connectivity in the intraparietal sulcus, frontal eye fields, and motion sensitive area (V5) [13]. Therefore, differences in neural mechanisms and activities between anisometropic and strabismic amblyopia could be an important source of bias in clinical studies on amblyopia, since most of them use a heterogenic sample.

Conventional treatments for amblyopia are glasses, patch, and penalization with atropine drops or Bangerter filters, but in recent years new approaches based on computerized visual training using different types of stimuli have been developed and evaluated. These trainings are justified by the influence of video games in neuro-modulatory pathways and the enhancement of attentional skills promoted by these games according to neurophysiological studies [14]. These new approaches have allowed clinicians to develop new protocols based on the following techniques: perceptual learning, dichoptic training, and binocular therapy. Perceptual learning consists of the stimulation of the visual pathway with Gabor's stimuli through the repetition of perceptual visual tasks [5, 15, 16], promoting an improvement in visual acuity (VA) and contrast sensitivity (CS) in amblyopic eyes. Dichoptic training is normally based on the use of polarized glasses, whereas for binocular therapy the use of red–green glasses is required. With both techniques, binocular fusion training is performed using stimuli with some common parts and disparate elements for each eye individually [17, 18]. Specifically, active visual therapy based on perceptual learning, dichoptic stimulation, and binocular training with anaglyph glasses is an interesting new area of research that can complement and optimize conventional methods for amblyopia treatment [18, 19].

The aim of this article was to gather all the scientific literature about the effectiveness of vision therapy in children and teenagers with anisometropic amblyopia and analyze the quality of such scientific evidence. For that purpose, a systematic review was performed, which is an exhaustive search that follows a strict protocol, uses several databases, and adds an analysis of the quality of the articles. Therefore, systematic reviews are the best option to add high-level quality for an evidence-based clinical practice.

## 2. Methods

A search was conducted using three searching strategies (Table 1) in 4 different databases: PubMed, Web of Science, Scopus, and ProQuest. Both types of amblyopia, strabismic and anisometropic, and all ages were included in the search to avoid missing relevant articles. Afterward, a refined selection of the articles was performed following these criteria:

- Original articles whose aim was to evaluate active visual therapy treatments for anisometropic amblyopia based on perceptual learning, dichoptic therapy, video games, software, binocular exercises, virtual reality, orthoptics, pleoptics, and any other active therapy procedures
- Randomized clinical trials (RCTs) and nonrandomized studies of intervention (NRSI)
- Population until 18 years old with anisometropic amblyopia
- Articles in English or Spanish
- Articles since 2008

Article selection was carried out in sequential steps. First, titles and abstracts were reviewed for excluding those which were not relevant for this study; next, duplicates were excluded. Second, complete texts were reviewed, selecting only those documents which comply with the previously defined criteria and answering our research question. Third, manual search was done to obtain references that might have not appeared during the first step. Articles with strabismic and anisometropic amblyopia that did not clearly sort the results by type of amblyopia were excluded.

Finally, for quality evidence assessment, two tools for risk of bias assessment recommended by Cochrane organization were used: ROBINS-I for nonrandomized studies of intervention (NRSI) [20] and ROB 2.0 for randomized clinical trial [17]. Both tools are divided into domains that analyze the main aspects of the articles, such as interventions, participants' characteristics, data collection, or deviations from the intended intervention. Following the guidelines of each evaluation tool, a table summarizing the quality of every article revised based on the risk of bias was obtained.

The first documentary search was carried out in November 2018, and databases were reviewed again in June 2019 applying the same method.

## 3. Results

### 3.1. Search Results

**Figure 1:**
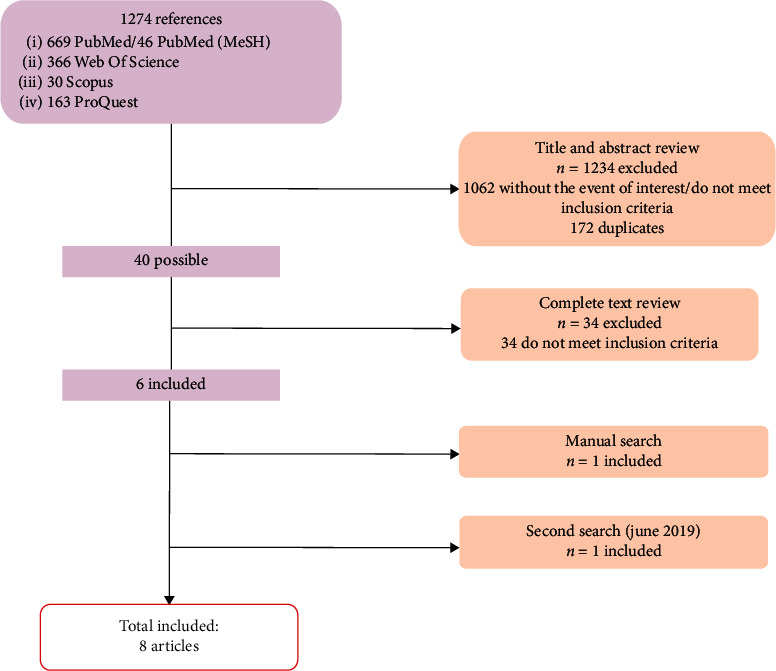
Flowchart showing the procedure followed during the systematic review.

Initially, a total of 1274 documents were obtained in the search. After reviewing titles and abstract and dismissing duplicates, only 217 articles were included for complete text reading. Two hundred and eleven out of 217 of those articles were excluded because they did not meet the inclusion criteria. Finally, the six remaining articles were included. Manual search and a second search were carried out, obtaining two new suitable references (Figure 1).

### 3.2. Included Studies

The main aspects of the 8 included studies are summarized in Table 2. Also, assessments of risk of bias with ROB 2.0 and ROBINS-I are described in Tables 3 and 4. We found 2 RCTs with a proper risk of bias, and 5 of 6 NRSI with serious risk of bias. The main source of risk of bias for NRSI was the domain related to the measurement of the outcomes (domain 6).

### 3.3. Excluded Studies

Thirty-four articles were excluded after complete text review. Excluding reasons are described in Table 5. The main reason for this exclusion was that most of the studies did not clearly sort the results by type of amblyopia.

### 3.4. Effect of the Intervention

It cannot be calculated since neither RCTs nor NRSI showed relative risk values. For this reason, a meta-analysis could not be performed.

## 4. Discussion

According to the results of this systematic review, three main aspects should be analyzed in studies evaluating the effect of active vision therapy in amblyopia due to its importance in daily practice: visual acuity improvement, dose-response ratio, and adherence to treatment. Active visual therapy with dichoptic therapy, perceptual learning, anaglyph glasses, or some specific video games is effective in the treatment of anisometropic amblyopia to improve visual acuity [21–28] when it is compared with only glasses [24, 26], patching [23, 28], or placebo [21]. According to some authors, this improvement seems to be similar to patching [24, 26, 27], without a clear evidence confirming that vision therapy is more or less effective than patching for the treatment of anisometropic amblyopia in children. This suggests that the dose-response relationship should be considered as a second significant feature when evaluating the usefulness of visual therapy in amblyopia. According to the literature revised, patching has a linear dose-response curve and needs about between 178 and 276 hours to gain 0.2 logMAR in children [29, 30], while visual therapy seems to require between 10 and 20 hours [27] in children for the same improvement in visual acuity. In any case, it should be noted that age is an important variable to consider because younger children need shorter treatments. Therefore, vision therapy seems to be at least as effective as patching and reduces processing time [25]. Furthermore, when combined with patching, vision therapy tends to provide even better results than the use of only patching. For instance, Singh et al. reported significant differences in their RCT, since the group that received dichoptic therapy and patching improved 2.4 lines in VA, while the patching group enhanced 1.8 lines [23]. In addition, good treatment compliance (mean of 69% or more) with visual therapy was reported by many authors [24–28], except a rate of 50% of compliance reported by Birch et al. [21]. This is an important advantage compared to patching, as its compliance ranges from 44% to 57% [31].

Concerning other important aspects such as stereopsis, regression, and contrast sensitivity, there is not enough scientific evidence to extract consistent conclusions. Stereopsis did not experience a significant improvement with vision therapy according to the scientific studies revised, but it should be investigated further, as it was only measured in 3 of 8 studies [21, 23, 27]. This fact may be due to several factors, one of them being the lack – until recently –of specific exercises for stereoacuity training, something which has been improved and optimized in recent years. For example, Portela-Camino et al. and [32] Kelly et al. [33] recently reported a significant improvement in stereopsis in amblyopic children after vision therapy, and even similar results were found in a prospective experiment performed by Ziak et al. where 17 adults with anisometropic amblyopia received dichoptic therapy using a virtual reality head-mounted display [34]. Future studies might add more knowledge about how stereopsis infers the recovery of anisometropic amblyopia and the time of treatment. Regarding visual acuity regressions, no significant cases have been reported, although only 2 of 8 studies described this issue and the analysis reported in such studies was in the short term [25, 27]. Contrast sensitivity was only measured in one study, reporting a nonsignificant improvement after therapy [23]. Finally, no adverse events during or after vision therapy have been described in the revised articles.

In addition to that, the lack of homogeneity of the protocols should be pointed out. As can be observed in the studies, frequency and duration of the vision therapy sessions differ among authors. For example, Singh et al. [23] and Totsuka et al. [28] prescribed 1 hour of training per day, Deshpande et al. [25] up to 2 hours per day, and Kuruca et al. [22] 15 minutes per day 6 times a week. While Birch et al. [21] prescribed 4 hours per week, Iwata et al. [24, 26] prescribed 2 sessions per week (30 minutes per day), and Gambacorta et al. prescribed 20 hours of training with no specific time per day [27]. Furthermore, some authors also combined vision therapy with near task and patching, but not all of them. Consequently, results can be biased by the differences in the frequency and duration of the vision therapy. Apart from that, when amblyopia is accompanied by strabismus, protocols are also different from anisometropic amblyopia. First, monocular treatment of amblyopia is focused on the recovery of visual acuity, and second, prism or strabismus surgery is also needed to obtain bifoveal fixation [35, 36] before binocular treatment of suppression with dichoptic therapy. Therefore, there is an important need of accordance on how training sessions and treatment protocols should be prescribed in amblyopia.

Regarding the quality of the articles revised, some features should be considered for understanding the results of the studies. RCTs were assessed by ROB 2.0, which showed an acceptable risk of bias in both articles. However, ROBINS-I for the revised NRSI showed that 5 of 6 articles had a serious risk of bias. Therefore, evidence of quality does not seem to be entirely acceptable and results of this review should be considered carefully for daily practice. The main weakness observed in NRSI was in domain 6 (measurements of outcomes) because there were no studies using an independent examiner for measuring visual acuity and a different one for prescribing and applying the treatment. Moreover, there is only one article with a 1-year follow-up, while the rest of the studies revised only reported outcomes in a short-term. Consequently, there is a need for double-blind trials with longer follow-up periods which provide better quality scientific evidence on the efficacy of vision therapy in amblyopia. This fact entails one of the most difficult goals in amblyopia research due to the following: RCTs in vision therapy require an appropriate sample of subjects who meet the inclusion criteria with a long-term follow-up, which is difficult to obtain due to the low prevalence of amblyopia; and it is even harder if the sample is selected by the type of amblyopia, as this review recommended. Furthermore, there are no double-blinded studies which specify that the examiner who did the examination before and after the vision therapy is not the same person who controlled the treatment. Above all, RCTs require a placebo, which should be properly designed and validated before the experiment, which means more time and investment.

Some issues should be considered to properly understand this systematic review. First, this review adds scientific evidence about new treatment approaches of amblyopia with vision therapy, which is the less studied treatment when compared with patching and optical prescription [30, 37–39]. It also emphasizes how essential it is to discern amblyopia according to its type in the studies, since there are reported differences between strabismic, mixed, and anisometropic amblyopia which can be a source of bias. These facts are the main strengths of this review because most of the previous articles (see Table 5) do not differentiate results by type of amblyopia when vision therapy is applied. Tailor et al. [40] still mentioned this issue when only 1 of 9 NRSI of their systematic review about vision therapy in amblyopia used anisometropic amblyopes. And second, the aforementioned lack of standardized protocols of vision therapy and well-performed RCTs entail an important limitation in evidence-based clinical practice, which should be resolved with further research.

In conclusion, active vision therapy is a promising option for the treatment of anisometropic amblyopia in children. However, there is still limited scientific literature concerning this issue with high levels of quality. Therefore, further research is needed to improve knowledge about the effectiveness of treatment protocols with vision therapy in amblyopia and to determine which neural mechanisms are specifically involved. A combined treatment of vision therapy and patching is a potentially more adequate treatment option for anisometropic amblyopia, allowing the clinician to optimize the processing time, minimize the psychosocial impact due to a prolonged patch wearing, improve the adherence to treatment, and address more visual skills than only visual acuity. This is something that should be investigated further in future RCTs with more strict inclusion criteria and methodology.

## Figures and Tables

**Table 1:** Search strategies.

*Strategy 1: free language*	

#1	Visual therapy
#2	Visual rehabilitation
#3	Dichoptic
#4	Dichoptic visual therapy
#5	Perceptual learning
#6	Pleoptics
#7	Software
#8	Video games
#9	Computer games
#10	Virtual reality
#11	VR
#12	Orthoptics
#13	#1 OR #2 OR #3 OR #4 OR #5 OR #6 OR #7 OR #8 OR #9 OR #10 OR #11 OR #12
#14	Amblyopia
#15	Anisometropic amblyopia
#16	Strabismic amblyopia
#17	Lazy eye
#18	Interocular suppression
#19	#14 OR #15 OR #16 OR #17 OR #18
#20	Child
#21	Children
#22	Childhood
#23	Young
#24	Youth
#25	Adults
#26	Elder
#27	Senior aged
#28	Preschool
#29	#20 OR #21 OR #22 OR #23 OR #24 OR #25 OR #26 OR #27 OR #28
#30	Visual acuity
#31	VA
#32	Stereopsis
#33	Contrast sensitivity
#34	#30 OR #31 OR #32 OR #33
#35	#13 AND #19 AND #29 AND #34

*Strategy 2: controlled vocabulary (MeSH terms)*	

#1	“Software” [mesh]
#2	“Video games” [mesh]
#3	“Virtual reality” [mesh]
#4	“Virtual reality exposure therapy” [mesh]
#5	“Orthoptics” [mesh]
#6	#1 OR #2 OR #3 OR #4 OR #5
#7	“Amblyopia” [mesh]
#8	“Child” [mesh]
#9	“Young adult” [mesh]
#10	“Adolescent” [mesh]
#11	“Adult” [mesh]
#12	“Aged” [mesh]
#13	#8 OR #9 OR #10 OR #11 OR #12
#14	“Visual acuity” [mesh]
#15	“Depth perception” [mesh]
#16	“Contrast sensitivity” [mesh]
#17	#14 OR #15 OR #16
#18	#6 AND #7 AND #13 AND #17

*Strategy 3: free language search used in scopus*	

#1	Visual
#2	Therapy
#3	#1 AND #2
#4	Visual
#5	Rehabilitation
#6	#4 AND #5
#7	Dichoptic
#8	Visual
#9	Therapy
#10	#7 AND #8 AND #9
#11	Perceptual
#12	Learning
#13	#12 AND #13
#14	Pleoptics
#15	Software
#16	Video games
#17	Computer
#18	Game
#19	#18 AND #19
#20	Virtual
#21	Reality
#22	#20 AND #21
#23	VR
#24	Orthoptics
#25	#3 OR #6 OR #10 OR #13 OR #14 OR #15 OR #16 OR #19 OR #22 OR #23 OR #24
#26	Amblyopia
#27	Anisometropic
#28	Amblyopia
#29	#27 AND #28
#30	Strabismic
#31	Amblyopia
#32	#30 AND #31
#33	Lazy
#34	Eye
#35	#33 AND #34
#36	Interocular
#37	Suppression
#38	#36 AND #37
#39	#26 OR #29 OR #32 OR #35 OR #38
#40	Child
#41	Children
#42	Childhood
#43	Young
#44	Youth
#45	Adults
#46	Elder
#47	Senior
#48	Aged
#49	Preschool
#50	#40 OR #41 OR #42 OR #43 OR #44 OR #45 OR #46 OR #47 OR #48 OR #49
#51	Visual
#52	Acuity
#53	#51 AND #52
#54	VA
#55	Stereopsis
#56	Contrast
#57	Sensitivity
#58	#57 AND #58
#59	#53 OR #54 OR #55 OR #58
#60	#25 AND #39 AND #50 AND #59

**Table 2:** Main aspect of the included studies.

Author (year)	Study design	Intervention	*n*	Age (years)	Follow-up	Conclusions
Totsuka (2018)	NRSI	Dichoptic therapy (Occlu-pad)	72 (35 aniso)	3–9	12 months	The Occlu-pad group showed greater improvement from the 6th month onward (*p* < 0.05)
		Patch	66 (35 aniso)	3–9	12 months	Adherence was better in the Occlu-pad group than the patching group (70%–34%)

Deshpande (2018)	NRSI	Perceptual learning + patch	32	8–12	3 months	All subject showed total or partial improvement (3 lines) in VA
		Perceptual learning + patch	18	13–20	3 months	Better effect at 3 and 4 weeks

Iwata (2018)	NRSI	Dichoptic therapy (Occlu-pad) + glasses	22	4.7 ± 1.2	6 months	Significant VA improvement (*p* < 0.05) at 3 and 6 months
						Good adherence to treatment (88.6% ± 18.9% under 3 months and 73.2% ± 18.9% between 3 and 6 months)

Gambacorta (2018)	NRSI	Dichoptic therapy	13			DT was better than PL but with no significance (*p* > 0.05). Likely due to the size of the sample
		Perceptual learning	16	7–17	Variable	Anisometropic amblyopia improved 0.1 ± 0.03 logMAR after 10 h of training (DT or PL)
						Similar results than patching. Shorter treatment time with DT and PL
						Compliance of at least 69%

Kuruca (2015)	NRSI	Perceptual learning (CAM)-anisometropic	15	4–10	6 months	Significant VA improvement in anisometropic amblyopes (*p* < 0.05)
		Perceptual learning (CAM)-strabismic	14	4–10	6 months	

Birch (2015)	NRSI	R-G glasses + iPad videogame	45	3.7–6.9	3 months	iPad videogame was better than sham iPad videogame
		Sham iPad videogame (placebo)	5	3.7–6.9	3 months	Significant VA improvement (*p* < 0.05)
						Compliance of at least 50%

Iwata (2018)	RCT	Glasses	23	3–8	6 months	Occlu-pad and glasses were better than only glasses (*p* < 0.05)
		Dichoptic therapy (occlu-pad) + glasses	23	3–8		Good adherence to Occlu-pad treatment (88.4 ± 18.7%)
						Similar outcomes than patching

Singh (2017)	RCT	Monocular videogame + patch	34	6–14	3 months	Monocular videogame and patch showed better results than only patch (2.4 lines logMAR/1.8 lines logMAR, *p* < 0.05)
		Patch	34	6–14	3 months	

PL = perceptual learning, RCTs = randomized control trials, VA = visual acuity, DT = dichoptic therapy, NRSI = nonrandomized studies of intervention.

**Table 3:** Results of ROB 2.0 tool for risk of bias assessment.

Author (year)	Domain 1: randomization process	Domain 2: risk of bias due to deviations from the intended interventions	Domain 3: missing outcome data	Domain 4: measurement of the outcome	Domain 5: selection of the reported result	Overall
Iwata (2018)	Some concerns	Some concerns	Low risk	Low risk	Low risk	Some concerns

Singh (2017)	Low risk	Low risk	Low risk	Low risk	Low risk	Low risk

**Table 4:** Results of ROBINS-I tool for risk of bias assessment.

Author (year)	Domain 1: confounding	Domain 2: selection of participants into the study	Domain 3: classification of the interventions	Domain 4: deviations from the intended interventions	Domain 5: missing data	Domain 6: measurements of outcomes	Domain 7: selection of the reported results	Overall
Deshpande (2018)	Low risk	Low risk	Low risk	Low risk	Low risk	Serious risk	Low risk	Serious risk
Iwata (2018)	Low risk	Low risk	Low risk	Low risk	Low risk	Serious risk	Low risk	Serious risk
Kuruca (2015)	Low risk	Low risk	Low risk	Low risk	Low risk	Serious risk	Low risk	Serious risk
Birch (2015)	Low risk	Low risk	Low risk	Moderate risk	Low risk	Moderate risk	Moderate risk	Moderate risk
Gambacorta (2018)	Low risk	Low risk	Low risk	Low risk	Moderate risk	Serious risk	Low risk	Serious risk
Totsuka (2018)	Low risk	Low risk	Low risk	Low risk	Moderate risk	Serious risk	Low risk	Serious risk

**Table 5:** Excluded articles in the systematic review.

Author (year)	Excluding reason
Lee et al. (2018)	Do not clearly sort the results by type of amblyopia
Portela et al. (2018)	Do not clearly sort the results by type of amblyopia
Kelly et al. (2018)	Do not clearly sort the results by type of amblyopia
Mezad-Koursh et al. (2018)	Do not clearly sort the results by type of amblyopia
Manh et al. (2018)	Do not clearly sort the results by type of amblyopia
Gao et al. (2018)	Do not clearly sort the results by type of amblyopia
Gao et al. (2018)	Do not clearly sort the results by type of amblyopia
Hamm et al. (2017)	The study includes anisometropic, strabismic, and deprivation amblyopia and those that do not sort the results by type of amblyopia
Barollo et al. (2017)	Is not a RCT or a NRSI
Bossi et al. (2017)	This study does not meet the inclusion criteria because only 7 of 22 children are anisometropic amblyopes and there is no control group, so we classified the study as case series
Dadeya et al. (2016)	Do not clearly sort the results by type of amblyopia
Kelly et al. (2016)	Do not clearly sort the results by type of amblyopia
Rajavi et al. (2016)	This study includes strabism until 10 diopters of deviation and do not sort the results by type of amblyopia
Holmes et al. (2016)	Do not clearly sort the results by type of amblyopia
Guo et al. (2016)	This study is an ongoing trial. In addition, it does not clearly sort the results by type of amblyopia
Webber et al. (2016)	Do not clearly sort the results by type of amblyopia
Herbison et al. (2016)	Do not clearly sort the results by type of amblyopia
Erbagci et al. (2015)	This study does not meet the inclusion criteria because authors do not use active visual therapy
Moseley et al. (2015)	This study does not meet the inclusion criteria because authors do not use active visual therapy
Hussain et al. (2014)	Only one child in the study has anisometropic amblyopia
Li et al. (2014)	This study includes strabismic children previously treated with glasses or surgery
Mansouri et al. (2014)	Do not clearly sort the results by type of amblyopia. Adults are included in the analysis
Herbison et al. (2013)	Do not clearly sort the results of the analysis by type of amblyopia. Only four children are anisometropic amblyopes
Foss et al. (2013)	Do not clearly sort the results by type of amblyopia
Lyon et al. (2013)	The objective of this study was to assess the adherence to treatment. There are no results about efficacy
Zhang et al. (2013)	This study does not meet the inclusion criteria because it is a retrospective study
Tijam et al. (2012)	Do not clearly sort the results by type of amblyopia
Knox et al. (2012)	Only two children are anisometropic amblyopes
Liu et al. (2011)	Do not clearly sort the results by type of amblyopia
Evans et al. (2011)	Do not clearly sort the results neither by type of amblyopia nor age
Wu et al. (2010)	This study does not meet the inclusion criteria. Authors do not use active visual therapy
Polat et al. (2009)	This study does not meet the inclusion criteria. It is a pilot study where 2 of 5 subjects have strabismus
Cleary et al. (2009)	Do not clearly sort the results by type of amblyopia
Awan et al. (2009)	This study does not meet the inclusion criteria because it is a retrospective study
